# Acute-Hypoxemia-Induced Right-To-Left Shunting in the Presence of Patent Foramen Ovale

**DOI:** 10.7759/cureus.16138

**Published:** 2021-07-03

**Authors:** Muhammad Atif Masood Noori, Abanoub Rushdy, Kalpesh k Shah, Fayez Shamoon, Mohammad Naser

**Affiliations:** 1 Internal Medicine, Rutgers New Jersey Medical School, Trinitas Regional Medical Center, Elizabeth, USA; 2 Cardiology, Saint Joseph's University Medical Center, Paterson, USA

**Keywords:** hypoxemia, right to left shunting, pulmonary hypertension, rv hypokinesia, atrial fibrillation, patent foramen ovale

## Abstract

Patent foramen ovale (PFO) is a common congenital abnormality of the heart. It results from incomplete closure of foramen ovale that persists in adulthood. Most individuals with PFO are asymptomatic and are discovered incidentally. The left atrial pressure is generally higher than the right atrial pressure, which prevents blood flow against the gradient; however, any medical condition that increases the pulmonary artery pressure can lead to reversal of blood flow from right to left by elevating right atrial pressure. We present a case of a 59-year-old female who presented with complaints of shortness of breath associated with bilateral lower-extremity edema and was found to have acute decompensated heart failure and atrial fibrillation. Transesophageal echocardiogram (TEE) with cardioversion was performed. Propofol was given for conscious sedation; however, the procedure was terminated as patient became hypoxemic and was noted to have moderately dilated right ventricle (RV) with hypokinesia and PFO with right-to-left shunting. It also demonstrated mild mitral regurgitation, mild left ventricular hypertrophy, and a left ventricular ejection fraction of 55-60%. In contrast to TEE findings, while the patient was having normal oxygen saturation, transthoracic echocardiogram showed left-to-right shunting instead of right-to-left and no RV hypokinesia was noted. In conclusion, this case draws attention to the relationship between acute hypoxemia and right-to-left shunting in a patient with PFO. This case illustrates and highlights the need for more prospective studies to establish a relationship between acute hypoxemia and right-to-left shunting in the presence of PFO.

## Introduction

Patent foramen ovale (PFO) is a common congenital abnormality of the heart. It results from incomplete closure of foramen ovale that persists in adulthood [1]. Most individuals with PFO are asymptomatic and are discovered incidentally, but PFO can be associated with serious clinical consequences, including cryptogenic stroke, decompression sickness in scuba divers, platypnea-orthodeoxia syndrome, and migraine with aura [1-3]. The left atrial pressure is generally higher than the right atrial pressure, which prevents blood flow against the gradient. We present the case of a 59-year-old female with no known past medical history who was noted to have acute-hypoxemia-induced right-to-left shunting in the presence of PFO while undergoing transesophageal echocardiogram (TEE). 

## Case presentation

**Figure 1:**
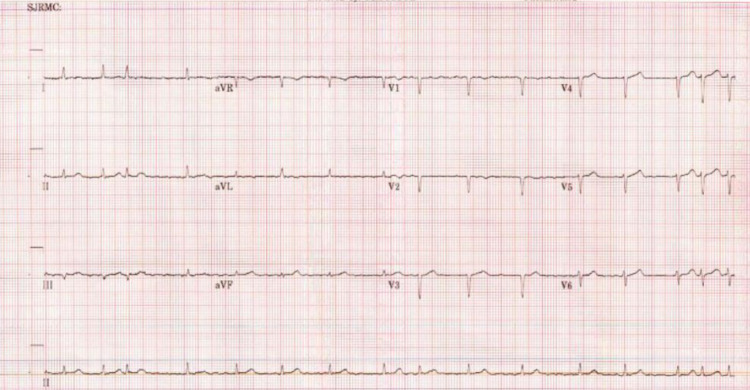
An ECG shows atrial fibrillation, low-voltage QRS complex with possible anteroseptal infarct

A fifty-nine-year-old female with no known past medical history came to the ED with complaints of shortness of breath associated with bilateral lower-extremity edema for three days. She had no past medical history of hypertension, diabetes mellitus, coronary artery disease, and no significant family history. She did not smoke cigarette (or tobacco) or consume alcohol. She denied any chest pain, cough, fever, orthopnea, paroxysmal nocturnal dyspnea, palpitation, or any recent immobilization. Patient never had similar symptoms before. Her blood pressure was 203/103 and heart rate was 92 with irregular rhythm. The patient was morbidly obese and moderately nourished. Chest auscultation revealed mild crackles bilaterally from mid to lower lung fields. Bilateral pitting edema was noted on lower extremities. An ECG demonstrated atrial fibrillation and low-voltage QRS complex ( Figure 1).

Chest X-ray showed bilateral pulmonary edema. Laboratory studies showed the following values: white blood cell count 9.5k/UL (4.8-10.8k/UL), creatinine 0.63 mg/dL (0.7-1.2 mg/dL), blood urea nitrogen 14 mg/dL (8-20 mg/dL), B-type natriuretic peptide 288 pg/mL (<100 pg/mL), troponin 0.01 ng/mL (<0.5 ng/mL). IV Lasix 40 mg daily and lisinopril 5 mg daily were started for acute decompensated heart failure. Therapeutic anticoagulation with Lovenox was also started. Later on the day of admission, the patient became tachycardic with heart rate hovering around 130-140. Telemetry showed atrial fibrillation with rapid ventricular response. For rate control, she received 20 mg IV Cardizem and got loaded with digoxin but given that she was persistently tachycardic, a decision was made to perform TEE with cardioversion. Propofol was given for conscious sedation. TEE was terminated after cardioversion to normal sinus rhythm as patient desaturated to 70s. TEE revealed moderately dilated right ventricle (RV)/RV hypokinesia, PFO with right-to-left shunting, mild mitral regurgitation, mild left ventricular hypertrophy, left ventricular ejection fraction (LVEF) 55-60%, and no evidence of left atrial thrombus (Video 1).

**Video 1:** Transesophageal echocardiogram shows right-to-left flow at patent foramen ovale with color flow mapping

Transthoracic echocardiogram (TTE) was also performed after TEE while she was saturating normal, and it showed an LVEF of 55-60%, mild left ventricular hypertrophy, moderately dilated left atrium, and a moderately elevated pulmonary artery systolic pressure at 43 mmHg, but in contrast to the results of TEE, left-to-right shunting instead of right-to-left was seen and no RV hypokinesia was noted (Video 2).

**Video 2:** Transthoracic echocardiogram shows left-to-right flow with color flow mapping.

Sotalol 120 mg BID was started for atrial fibrillation and a repeat synchronized cardioversion was performed for persistent atrial fibrillation. Patient had a STOP BANG score of 5, concerning for obstructive sleep apnea (OSA), and she was advised to follow up with pulmonology for sleep study.

## Discussion

This case report documents acute on chronic hypoxemia-induced right-to-left shunting secondary to worsening of prior pulmonary hypertension in the presence of PFO. 

Throughout the cardiac cycle, left atrial pressure is generally higher than the right atrial pressure, which prevents blood flow against the gradient; hence, PFO generally does not result in hypoxemia. Certain medical conditions (e.g. chronic obstructive pulmonary disease, OSA, and obesity hypoventilation syndrome) that increase the pulmonary artery pressure can lead to reversal of blood flow from right to left by elevating right atrial pressure, causing hypoxemia [4]. Our patient had suspected OSA with Grade 2 pulmonary hypertension. Shanoudy et al. described higher prevalence of PFO in patients with OSA [5]. Stefano et al. showed the improvement in nocturnal oxygenation and sleep-disordered breathing in OSA patients who had PFO closure [6].

Propofol is known to cause hypoxemia, especially in obese patients. Our patient had a significant drop in arterial oxygen saturation from 94% to 70% during TEE. Acute hypoxemia is believed to transiently elevate pulmonary artery pressure as a result of increased pulmonary arteriolar resistance, which in turn causes elevation of right-sided pressure. In the presence of a PFO, this increase in right-sided pressure opens up the foramen ovale flap, increasing right-to-left shunt and further worsening hypoxemia. Kathrine et al. showed a pig model of acute right ventricular after-load increase by hypoxic pulmonary vasoconstriction. The study demonstrated that mean pulmonary arterial pressure and pulmonary vascular resistance can be increased significantly by lowering FiO_2_ [7].

Our patient had acute-hypoxemia-induced RV hypokinesia leading to right-to-left shunting in the presence of PFO, which further exacerbated hypoxemia, as demonstrated by TEE but not seen in TTE that was performed two days later at the time of normal saturation. Instead, later, it showed left-to-right shunting. Though our patient had underlying pulmonary hypertension probably secondary to suspected OSA, it is assumed that acute hypoxemia in the presence of chronic pulmonary hypertension can transiently elevate pulmonary artery pressure due to arteriolar vasoconstriction and lower the threshold of RV hypokinesia, causing right-to-left shunting.

## Conclusions

This case highlights the changes in hemodynamics expected with lower venous return, increased RV wall stress, and lower pulmonary compliance in patients with elevated systolic pulmonary vascular resistance. It adds to the available body of literature in this field the instant documentation of right-to-left flow of blood through PFO. This case further draws attention to the relationship between acute hypoxemia and right-to-left shunting in a patient with PFO. It would be interesting to see more articles examining this relationship further.
